# The multidisciplinary treatment of osteosarcoma of the proximal tibia: a retrospective study

**DOI:** 10.1186/s12891-018-2245-x

**Published:** 2018-09-05

**Authors:** Junqi Huang, Wenzhi Bi, Gang Han, Jinpeng Jia, Meng Xu, Wei Wang

**Affiliations:** 1 Department of Orthopaedics, Mianyang Central Hospital, Mianyang, 621000 Sichuan China; 2 Department of Orthopaedics, PLA General Hospital, Beijing, 100853 China

**Keywords:** Osteosarcoma, Tibia, Arthroplasty, Reconstruction, Survival

## Abstract

**Background:**

Survival and reconstruction constitute important challenges in multimodal treatment of osteosarcoma of the proximal tibia. The purpose of this study was to assess the efficacy and prognosis of neoadjuvant chemotherapy and custom-designed endoprosthetic arthroplasty.

**Methods:**

A total of 69 patients with osteosarcoma of the proximal tibia were evaluated, including 43 males and 26 females, treated with multidisciplinary limb-salvage remedy from October 2003 to December 2013. They were at least 12 years old (mean, 20 years; range, 12–57 years). The gap between tumor and main artery/nerve was showed in MRI. Mean follow up was 69.5 months (range, 9–144 months). Kaplan-Meier survival curves were generated to assess prognosis and relapse rate. The initial symptoms and disease duration for each patient were recorded. Correlation analyses were performed for the association of various parameters with prognosis. Functional outcomes were evaluated using the Musculoskeletal Tumor Society (MSTS) guidelines after 6 months postoperatively, to analyze the relation between bone excision size and function recovery.

**Results:**

The resection lengths measured intraoperatively ranged from 80 to 230 mm, and contained 3 cm of normal bone around the tumor. A total of 3 courses of preoperative chemotherapy were administered to all cases. At final follow-up, 1 case showed recurrence. Meanwhile, 8 patients (11.6%) died from lung metastasis. Post-operative infection occurred in 3 patients; 1 case was maintained with revision surgery. Two cases underwent amputation. The mean MSTS system score was 21.6.

**Conclusions:**

The multidisciplinary treatment result in an overall positive outcome, with improved function.

## Background

Osteosarcoma is a malignant tumor originated from a mesenchymal stem cell precursor that produces immature woven bone (osteoid) after becoming malignant [1]. It is the most common solid bone cancer, occurring in 2–3 per 106,000 individuals [2]. Previously, this disease was only treated by amputation; however, effective neoadjuvant/preoperative and postoperative adjuvant chemotherapy regimens allow safe limb-sparing resections, improving survival rates [3, 4]. Indeed, with the recent availability of multimodal treatment combining imaging, chemotherapy, and surgical techniques, 70–85% of malignant tumors are efficiently treated with limb salvage [5, 6], and long-term survival for patients with localized osteosarcoma now reaches approximately 60% [7, 8]. Limb-sparing surgery yields good oncological and functional outcomes, as well as satisfactory psychological results [9]. Despite multiple reports regarding the operative techniques, such as allografts and arthrodesis, the choice for reconstructing bone and soft-tissue defects after resection remains a serious challenge [10]. Meanwhile, complications after allografts reconstruction limited its application [11]. The arthrodesis had poor joint activity.

Prosthetic implantation has been proposed to result in physical improvement, but most individuals develop dismal femur lesions [11, 12]. According to any reports, overall survival is reduced due to recurrence [13, 14]. The proximal tibia is second only to distal femur in osteosarcoma frequency [5], with about 75% of all patients suffering from osteosarcoma around knee [15]. Unlike other sites, resection of osteosarcoma of the proximal tibia meeting the wide incision principle causes the loss of bone and patellar tendon; indeed, tibial growth plate will not return to normal after implantation of a distal femoral prosthesis [16, 17]. This likely increases the likelihood of relapse. In addition, periprosthetic-related accidents after surgery are numerous, including infections, aseptic loosening, wear of joint components, dislocations, prosthesis breakage, and fatigue; fractures are also common in the long run [18]. Interestingly, medial gastrocnemius rotational flap was shown to decrease soft tissue defects and the risk of infection [19]. We hypothesized that neoadjuvant chemotherapy combined custom designed prosthesis for osteosarcoma treatment would result in improved function and prolonged survival. Therefore, this study aimed to retrospectively assess the efficacy of neoadjuvant chemotherapy and custom-designed endoprosthetic arthroplasty for the treatment of osteosarcoma of the proximal tibia.

## Methods

### Study design

This retrospective cohort study assessed patients with osteosarcoma of the proximal tibia treated in our institution, between October 2003 and December 2013. It was approved by Ethics Committee of our hospital; informed consent was obtained from all patients.

### Patients

A total of sixty-nine patients who underwent neoadjuvant chemotherapy and hinge prosthesis, extensor function reconstruction of the proximal tibia after wide resection for osteosarcoma were assessed. There were forty-three males (62.3%) and twenty-six females (37.7%), averaging 20 years old (range, 12–57). The patients, diagnosed with stage IIB(Ennecking system, no metastasis) osteosarcoma by biopsy and imaging, were included if meeting the following eligibility criteria: complete neoadjuvant chemotherapy, no invasion of tibial artery and vein on imaging (MRI), no invasion of tibial (peroneal) nerve on imaging (MRI), no preoperative metastasis. Exclusion criteria included skin ulceration, tumor surrounding popliteal artery, metastasis showed on imaging. Data (e.g. Initial symptoms before treatment and their durations) were collected from medical records.

X-ray and MRI were taken before neoadjuvant chemotherapy and surgery. When imaging examination and laboratory examination were finished, biopsy was conducted in our institution. The pathologic report was confirmed by chief pathologist.

### Chemotherapy

Neoadjuvant chemotherapy was administered after positive pathology. Every cycle consisted of ifosfamide (2 g/m^2^/day on days 1 to 5) and doxorubicin (40 mg/m^2^/day on day 5). Based on physical condition and efficiency of multi-agents, additional drug was methotrexate 8 g/m^2^/day on day 3 (children) or cisplatin 120 mg/m^2^/day on day 6 (adults). Three and six courses, respectively, were administered preoperatively and postoperatively as standard therapy. Blood, liver function, renal function, and electrolyte assessment was performed during chemotherapy. About 1 month was allowed after every cycle. Complete standard therapy was executed unless patients showed intolerance.

### Surgery

All patients receiving 3 courses of chemotherapy were submitted to plain radiography, magnetic resonance imaging (MRI) and chest CT before surgery [20]. These tests determined the resection realm and prosthesis size. A rotary hinge endoprostheses was applied. An anteromedial incision started proximally at the distal third of the femur and extended below the lesion. The biopsy site was excised with a 3 cm margin. The medial sural artery supplying the medial gastrocnemius was preserved. The patellar ligament was detached 3 cm proximal to its insertion, and the knee capsule was incised 3 cm from tibial insertion. A specimen was collected 3 cm outside the normal tissue, based on T1 imaging data. The length of resection tumor was measured intraoperatively. It determined to choose the same standard prosthesis. An artificial joint was implanted using the cemented technique for component insertion. The extensor mechanism was advanced, and the remaining patellar tendon attached to the prosthesis where had immobilized groove. An allograft like LARS ligament was used to prolong tendon when residual patellar tendon had not sufficient to insert in prosthetic fixed groove. In case the muscle and deep fascia did not inadequately cover the prosthesis after wide resection, a medial gastrocnemius rotation flap would repair the passive spacer. Antibiotic was infused for a week after surgery.

### Postoperative care, indicators and follow up

The affected extremity flexion was kept slightly moist, with a drain left until the fluid is minimal. Antibiotics were routinely administered 1 week postoperatively. Knee extension was kept for 3 weeks to allow healing of knee extensor reconstruction. Active and active-assisted exercises were then encouraged, with the purpose to recover the range of motion and strength. Regarding the time of arthroplasty, the Musculoskeletal Tumor Society (MSTS) 93 scoring system was applied to comparably assess the muscle function 6 months postoperatively through outpatient review. Follow up was performed in outpatient service and by telephone. The **indices assessed** were pain, range of motion, emotional acceptance, supports (brace, cane, and crutches), walking ability and gait. Lung CT and X-ray of the affected limb were reviewed in outpatient service every 6 months, to detect local control and distant metastasis.

### Statistics

Data were presented as mean ± standard deviation (SD) or percentage, as appropriate. Correlation analyses were performed to determine the associations of various parameters with prognosis (survival, recurrence and metastasis). Kaplan-Meier survival curves were generated to assess overall 3- and 5-year patient survival rates. The correlation between prosthesis length and rehabilitation function was also evaluated. Function scores were based on MSTS indication, with 30 as maximum score and normal function. Any complication was considered to be related to prosthesis performance.

## Results

### Baseline patient data

All the patients underwent en block resection confirmed by pathology postoperatively and custom-designed prostheses arthroplasty for reconstruction. The clinical information was uncovered in Table 1. Precisely, the patients included 62.3 and 37.7% male and female individuals, respectively. Initial symptoms were pain and swelling in 19 patients (27.5%), pain in 41 (59.4%), and swelling in 9 (13.1%). The symptoms had lasted for 5 ± 10 (ranging from 1 to 20) months. Metal on polyethylene locking mechanism was used in prostheses.

**Table 1:** Baseline characteristics of patients

Parameter	Value
Gender	
Male	43(62.3%)
Female	26(37.7%)
Initial symptom	
Pain+swelling	19(27.5%)
Pain	41(59.4%)
Swelling	9(13.1%)
Average symptom duration	5 ± 10 months
Average follow-up	69.5 ± 4.3 months
Prosthetic complication	3(infection) 2(ligament Breakage)

### Limb function

**Fig. 1:**
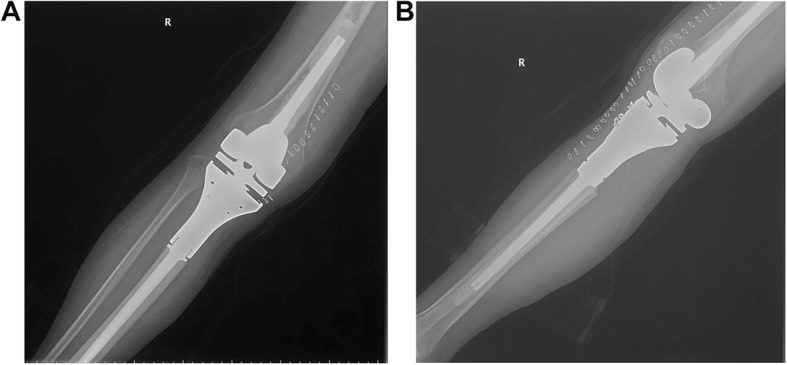
The graph shows front (**a**) and side (**b**) appearance of prosthesis postoperatively

Limb function was evaluated in all patients by the MSTS 93 system 6 months postoperatively [21]. A mean score of 21.6 was obtained, with values between 19 and 28. The range of knee motion was from 60°to 110°. 17 patients (24.6%) exercised to improve limb function 12 months postoperatively. The mean MSTS score was 22.3. The range of knee motion between 60°and 120°. Meanwhile, bone resection size was 13 cm (ranging 10 to 16 cm). Limb function was not associated with bone loss or exercise (*p* > 0.05 in correlation analyses). Figure 1 was the postoperative view of the prosthesis.

### Prognosis and survival

**Fig. 2:**
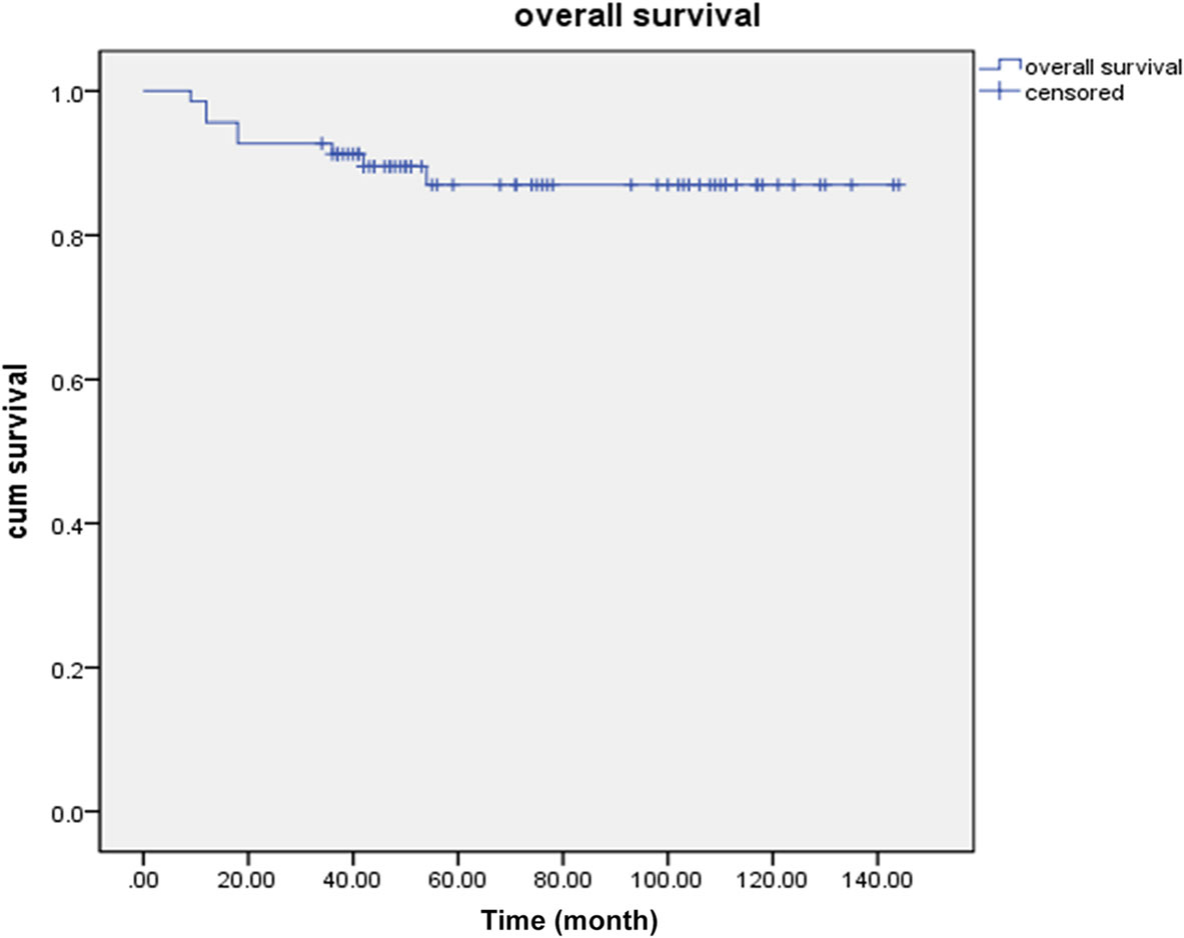
Overall survival was assessed by the by Kaplan-Meier method. The 3- and 5-year overall survival rates were 91.3% and 87%, respectively

Every patient was reexamined per 6 months. The examination contained limb X-ray, lung CT. Mean of follow up time was 75.9 months, with 95% confidence intervals between 67.4 and 84.4 months. At the last follow-up, 54 patients (78.3%) were continuously event-free, and 3 (6.5%) had evidence of infection; meanwhile, 8 patients (11.6%) had died of disease-related complications. The 3- and 5-year overall survival rates were 91.3% and 87%, respectively, as obtained by Kaplan-Meier analysis (Fig. 2). One case of local recurrence was observed after 5 months postoperatively; amputation was conducted and adjuvant chemotherapy added for two courses, and lung metastasis finally caused death 18 months after confirmed diagnosis. Correlation analyses indicated no significant associations of pain accompanying swelling or symptom duration before treatment with prognosis (both *p* > 0.05).

### Complications

Among the 3 cases with infection, 2 selected limb amputation for lack of money. The prosthesis was removed and bone cement containing vancomycin was implanted in 1 patient. Vancomycin and ceftriaxone were used when infection was confirmed. Revision surgery was conducted and drug withdrawal when infection indexes, such as C-reactive protein (CRP) and erythrocyte sedimentation rate (ESR) returned to normal. No infection was further verified by joint fluid culture. To date, no prosthesis-related disease has been recorded. Breakage of the wire fixing the patellar ligament occurred in 2 cases; this was removed and a second surgery was performed.

## Discussion

This study aimed to evaluate the efficacy of neoadjuvant chemotherapy and custom-designed endoprosthetic arthroplasty for treating osteosarcoma of the proximal tibia, and showed that the multidisciplinary treatment result in improved prognosis and survival.

It is critical to identify effective approaches for lesion treatment. With the introduction of chemotherapy, 5 year survival has dramatically improved in the last decades [22, 23], allowing limb salvage [22, 24]. However, the best association for neoadjuvant drug had not yet been confirmed. In the current study, we combined triplet regimen for increase of drug tolerance. The 5-year overall survival was 87%.

Prosthetic arthroplasty does not increase death and recurrence [25], providing an improved limb function [26]. According to the special anatomy of the proximal tibia, knee extensor mechanism reconstruction after resection is of utmost importance. Previous reports examined the artificial ligaments, osteotomized fibula, and autologous fasciae for reconstruction [27–29]. However, complications associated with proximal tibia prosthesis are common: it results in poor patellar tendon reattachment, infection, poor skin covering, mechanical wearing and loosening, and damage to neurovascular structures [10, 20, 30]. Based on en block resection, we preserved the normal patellar ligament for reattachment to the prosthesis by a wire. To minimize the incidence of loosening, synovitis, and trauma, allografts were selected in the combination method [31]. This reinforced the patellar tendon, and no case of poor reattachment was found in this retrospective study. When the preserved tendon was inadequate because of wide excision or excessive soft tissue removed from the anteromedial tibia, a medial gastrocnemius flap was employed to keep stretches stable and supply a comprehensive surrounding [32, 33], which shortened infection and promoted healing as observed during follow up.

Variable rates of infection in tumor endoprostheses have been reported [34, 35]. Operative time, blood loss and wound complications are risk factors for infection [36]. Despite conformation to asepsis, infection was found in 3 patients, as described above, probably due to immune suppression by chemotherapy and soft-tissue defects. The prosthesis was removed after infection was diagnosed, and antibiotic cement was placed as proposed previously [37]. Revision was not carried out until normal levels of blood infection markers were obtained. Of note, debridement and retention for management of early and late acute infections have significant success rates [36], likely providing an approach to resist microbial infection. Meanwhile, we found that infection incidence was plummeting among patients with medial gastrocnemius flap transposition.

No patient had aseptic loosening, whose incidence for proximal tibia reduced compared with that obtained for dismal femur. Indeed, mechanics indicated that the more pressure dismal femur prosthesis bears, the higher risk. Meanwhile, extensor mechanism in this study was stabilized with bone-muscle flap. The prosthesis matching the medullary space and enhanced cement promise low risk of loosening. Furthermore, other complications, such as injury of artery and nerve, were not found in this study; the reason might be that preoperative MRI clearly revealed anatomy around the knee. Breakage of prosthesis handle is mostly found among children [21], and fine handle to match the diameter of medullary increases the risk of breaking. In this study, mean patient age was 20 years. Preserving enough bone cortex and using thick handle overcame these shortcomings.

Independent exercising and rehabilitation were begun at postoperative week 3 in this study, with the purpose of ensuring ligament healing. For unifying function comparison at different intervention times, we selected 6 months post-surgery as MSTS assessment point. An average functional score of 21 was obtained, and normal flexion and extension were achieved by the majority of patients. Routine walking was achieved after muscle exercise. Bone loss affected muscle function slightly. The prosthesis had the ability to rebuild the defect, but MSTS values were lower than those of distal femur [38, 39]; this may be due to the involvement of patellar ligament reconstruction. Both local control and tissue reserve were crucial in method design.

This study has several limitations. First, this was a single study, with inherent selection bias. In addition to its small sample size, the patients were assessed retrospectively. Therefore, these findings should be confirmed in larger sample, multicenter, prospective studies.

## Conclusion

In summary, custom-designed endoprostheses, combined with neoadjuvant chemotherapy, result in an overall positive outcome, improving prognosis and survival. Therefore, the multidisciplinary treatment constituted an appropriate alternative in patients with osteosarcoma of the proximal tibia.
